# Single incision laparoscopic liver resection (SILL) – a systematic review

**DOI:** 10.3205/iprs000076

**Published:** 2015-12-15

**Authors:** Christian Benzing, Felix Krenzien, Georgi Atanasov, Daniel Seehofer, Robert Sucher, Ricardo Zorron, Johann Pratschke, Moritz Schmelzle

**Affiliations:** 1 Department of General, Visceral, and Transplant Surgery, Campus Virchow Klinikum and Department of General, Visceral, Vascular and Thoracic Surgery, Campus Mitte, Charité – Universitätsmedizin Berlin, Berlin, Germany

**Keywords:** single incision laparoscopic hepatectomy, single-site laparoscopic liver resection, single-port laparoscopic liver resection

## Abstract

**Background:** Today, minimally invasive liver resections for both benign and malignant tumors are routinely performed. Recently, some authors have described single incision laparoscopic liver resection (SILL) procedures. Since SILL is a relatively young branch of laparoscopy, we performed a systematic review of the current literature to collect data on feasibility, perioperative results and oncological outcome.

**Methods:** A literature research was performed on Medline for all studies that met the eligibility criteria. Titles and abstracts were screened by two authors independently. A study was included for review if consensus was obtained by discussion between the authors on the basis of predefined inclusion criteria. A thorough quality assessment of all included studies was performed. Data were analyzed and tabulated according to predefined outcome measures. Synthesis of the results was achieved by narrative review.

**Results:** A total of 15 eligible studies were identified among which there was one prospective cohort study and one randomized controlled trial comparing SILL to multi incision laparoscopic liver resection (MILL). The rest were retrospective case series with a maximum of 24 patients. All studies demonstrated convincing results with regards to feasibility, morbidity and mortality. The rate of wound complications and incisional hernia was low. The cosmetic results were good.

**Conclusions:** This is the first systematic review on SILL including prospective trials. The results of the existing studies reporting on SILL are favorable. However, a large body of scientific evidence on the field of SILL is missing, further randomized controlled studies are urgently needed.

## Zusammenfassung

**Hintergrund:** Laparoskopische Leberresektionen für gut- und bösartige Lebertumore werden heutzutage routinemäßig durchgeführt. In den letzten Jahren wurden darüber hinaus vermehrt laparoskopische Leberresektionen in Single-Port-Technik (SILL) durchgeführt. Da diese Technik insgesamt noch wenig etabliert ist, führten wir ein systematisches Review der aktuellen Literatur durch, um die Daten zu Machbarkeit und Grenzen der Prozedur, perioperativen Ergebnissen und onkologischen Resultaten zu sammeln.

**Methoden:** Es wurde eine Literaturrecherche mit vordefinierten Suchbegriffen auf Medline durchgeführt. Die Titel und Zusammenfassungen wurden unabhängig von zwei Autoren selektiert. Der Konsens, ob eine Studie in das Review eingeschlossen wurde, erfolgte nach Diskussion zwischen den Autoren auf der Basis prädefinierter Einschlusskriterien. Die Studien wurden zunächst hinsichtlich ihrer Qualitätsmerkmale bewertet, die Daten wurden anhand der zu untersuchenden Endpunkte gefiltert und tabellarisch dargestellt. Die Datensynthese erfolgte mittels eines narrativen Reviews.

**Ergebnisse:** Insgesamt wurden 15 Studien identifiziert, welche die Einschlusskriterien erfüllten. Hierunter war eine prospektive Kohortenstudie und eine randomisiert kontrollierte Studie, welche die SILL mit der konventionell laparoskopischen Leberresektion (MILL) verglich. Die übrigen Studien waren retrospektive Fallstudien mit maximal 24 Patienten. Alle Untersuchungen zeigten gute Resultate hinsichtlich kosmetischem Ergebnis, Machbarkeit, Morbidität und Mortalität. Die Rate an Wundkomplikationen und Narbenhernien war gering.

**Schlussfolgerungen:** Die Ergebnisse der aktuellen Studien zur SILL sind hervorragend. Allerdings fehlt eine breite Evidenz, um eine definitive Aussage über das Verfahren treffen zu können. Hierzu bedarf es weiterer klinischer randomisiert kontrollierter Studien.

## Introduction

Over the last four decades, laparoscopy has evolved from an experimental surgical approach into a well-established and standardized surgical method [1]. Since then, there were noticeable technical improvements. Nowadays, it is considered the golden standard for most abdominal surgical procedures [2]. The main advantage of laparoscopy compared to open surgery is a significant reduction of the abdominal trauma which leads to a faster postoperative recovery [3]. In classic laparoscopic surgery, at least three abdominal incisions for the corresponding trocars are needed. Recent scientific approaches have focused on reducing the number of ports needed. Besides the development of natural orifice translumenal endoscopic surgery (NOTES) [4], the introduction of single incision laparoscopic surgery (SILS) has led to a further reduction of abdominal incisions. The spectrum of surgical procedures that can be performed in SILS technique is broad [5], [6], [7], [8].

Despite these advancements in the field of laparoscopic surgery, liver surgery, especially in case of major hepatic resections, is still considered a domain of conventional open surgery. However, the laparoscopic resection of neoplasms of the left lateral liver [9], [10] or anterior inferior segments [11] has become standard. Moreover, major hepatic resections such as hemihepatectomies can be performed safely laparoscopically with good clinical results [12], [13]. Similar to other laparoscopic procedures, SILL reduces intraoperative blood loss, postoperative pain and are related to a faster recovery compared to an open surgical approach [14]. Recently, several reports about SILL have been published. Nonetheless, large clinical randomized trials and systematic reviews are missing. Thus, the benefit of these procedures remains uncertain.

The present review was designed to investigate the feasibility, perioperative results and oncological outcome of SILL procedures in adults compared to a conventional laparoscopic or open surgical approach.

## Methods

The present review was conducted according to the PRISMA guidelines for systematic reviews [15].

### Eligibility criteria

Studies that were considered for review met the following criteria:

- adult patients with SILL procedures
- original article including retrospective and prospective case series, prospective randomized controlled trials
- English language

### Literature search methodology

The literature research on Pubmed (MEDLINE) was rolled out on 21 November 2015 using MeSH keyword search. The search terms included “single incision laparoscopic liver resection”, “single-port laparoscopic liver resection”, “single site laparoscopic liver resection”, “laparoscopic liver resection”, “laparoscopic left lateral sectionectomy”. The articles that met the eligibility criteria were retrieved from the aforementioned database.

### Study selection

After the litereature research had been carried out, the list of available publications was screened by two reviewers. All studies that did not meet the eligibility criteria were excluded from the review process.

### Data items and synthesis

First, we determined the criteria for study quality (Table 1 (Tab. 1)) and study results (Table 2 (Tab. 2)). According to these factors, data extraction was independently performed by two reviewers, the results were noted on a standardized table.

The following outcome factors were analyzed:

histology, lesion size, radicality, oncolocigal long-term outcome, operative time, follow-up, length of hospital stay (LOS), blood loss, costs, scar length, mortality, conversion rate and perioperative complications (bile leakage, intraabdominal hematoma/abscess, incision hernia, wound infection, pleural effusion).

### Quality analysis 

The level of evidence was assessed according to the ASCO and ESMO gradation system [16]:

- Level I: Evidence based on meta-analyses of large controlled trials/large randomized controlled trials
- Level II: Evidence based on small randomized trials with uncertain results
- Level III: Evidence based on nonrandomized prospective case-control studies
- Level IV: Evidence based on nonrandomized historical cohort controls
- Level V: Evidence based on case series without controls

### Risk of bias

The risk of bias was assessed using a qualitative analysis based on the mentioned criteria on study quality and available data (Table 1 (Tab. 1) and Table 2 (Tab. 2)). Each study that was included in the review process was analyzed for detection or reporting bias. The assessment of bias was done according to the PRISMA guidelines [15].

## Results

### Study characteristics and risk of bias

**Figure 1:**
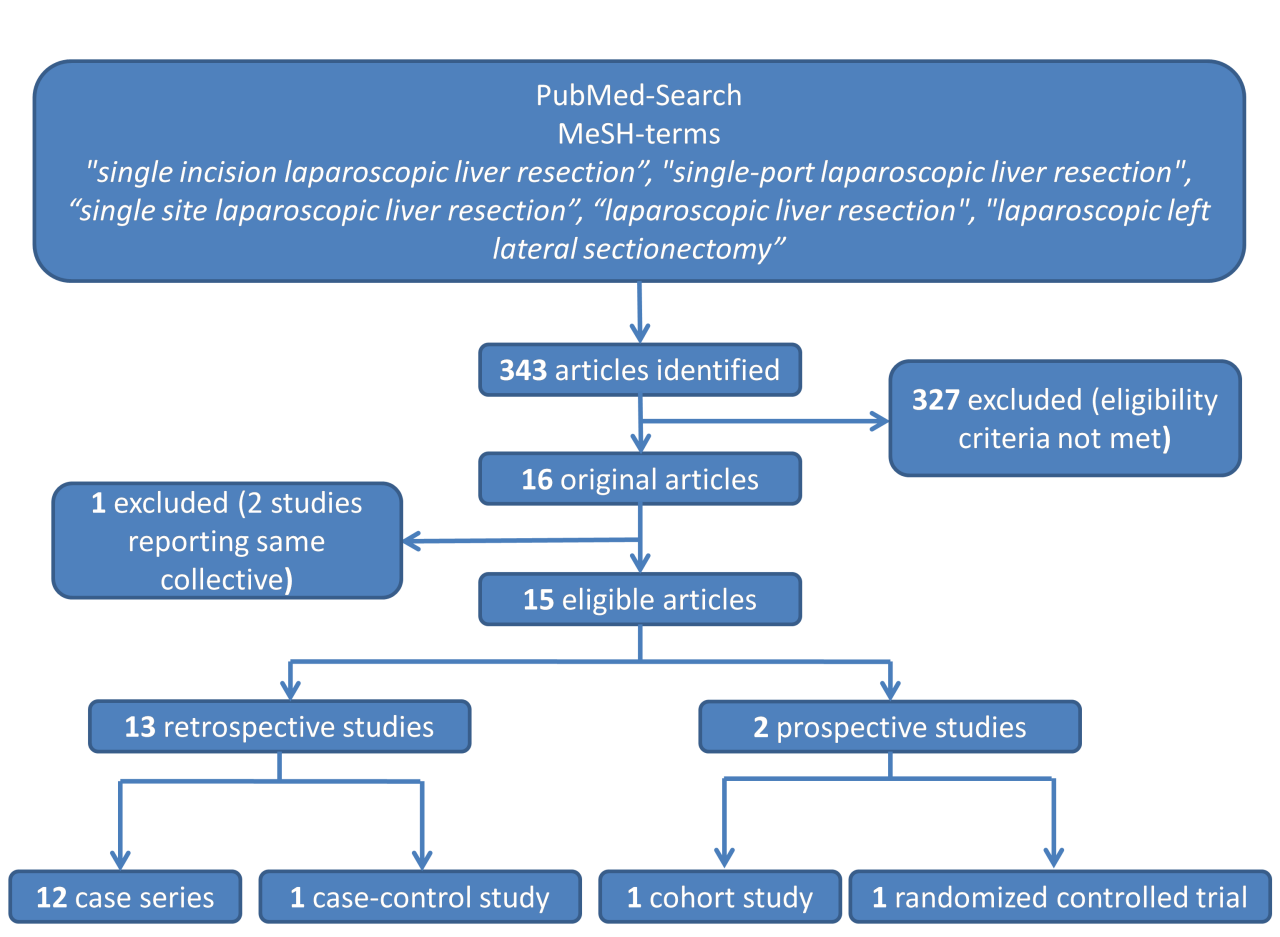
Overview of the search algorithm

The literature research revealed 343 articles, of which 16 [17], [18], [19], [20], [21], [22], [23], [24], [25], [26], [27], [28], [29], [30], [31], [32] met the inclusion criteria. One study [26] had to be excluded since the same patients were reported in another publication [27] (Figure 1 (Fig. 1)). Of these 15 eligible studies, there were 13 retrospective case series without control group (Level of evidence: V) [17], [18], [19], [20], [21], [23], [24], [25], [26], [29], [30], [31], [32]. In one case series a control group was available (conventional multiport laparoscopic approach; Level of evidence: IV) [27]. Of the two prospective trials, there was one cohort study [28] (Level of evidence: III) and one randomized controlled trial (Level of evidence: II) [22]. Table 1 (Tab. 1) provides an overview of the quality criteria of the studies.

### Patients and procedures

**Figure 2:**
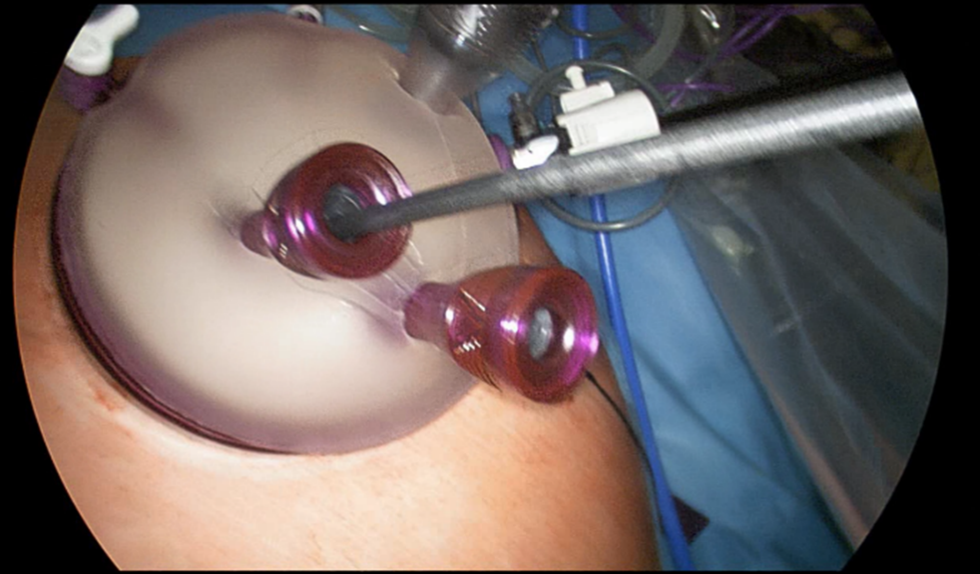
Placement of the Single-Port with four trocars

**Figure 3:**
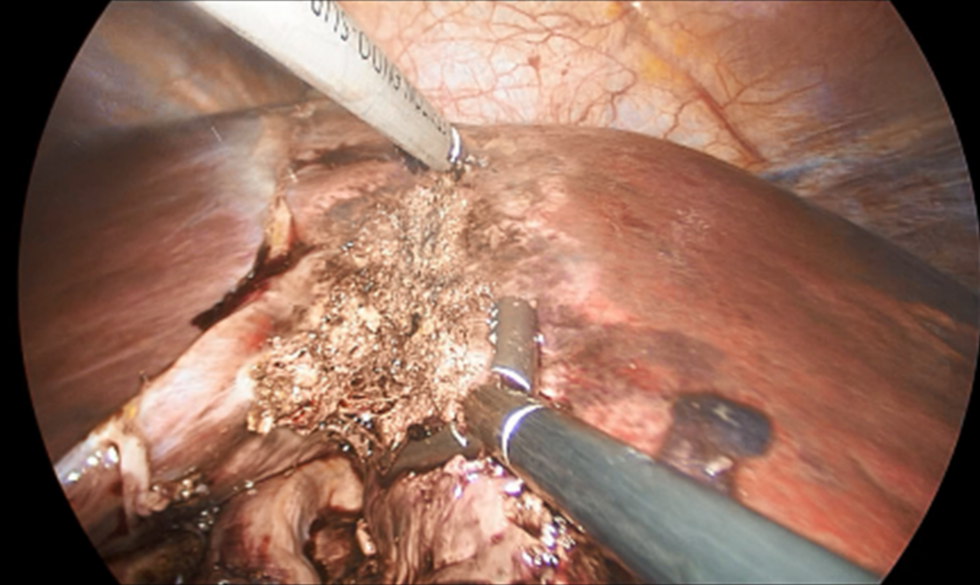
Dissection of the liver parenchyma using ultrasound scissors

**Figure 4:**
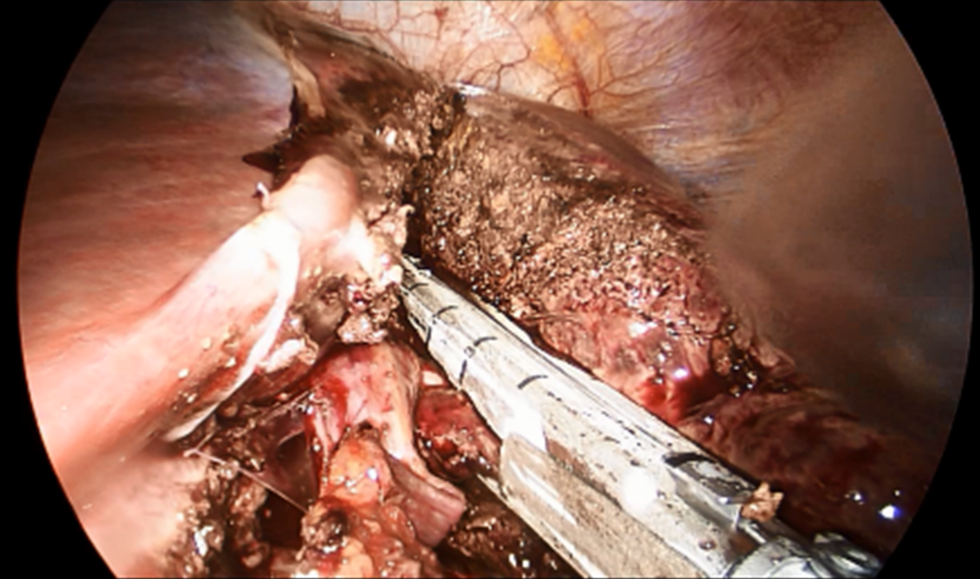
Dissection of the liver parenchyma using laparoscopic stapler

**Figure 5:**
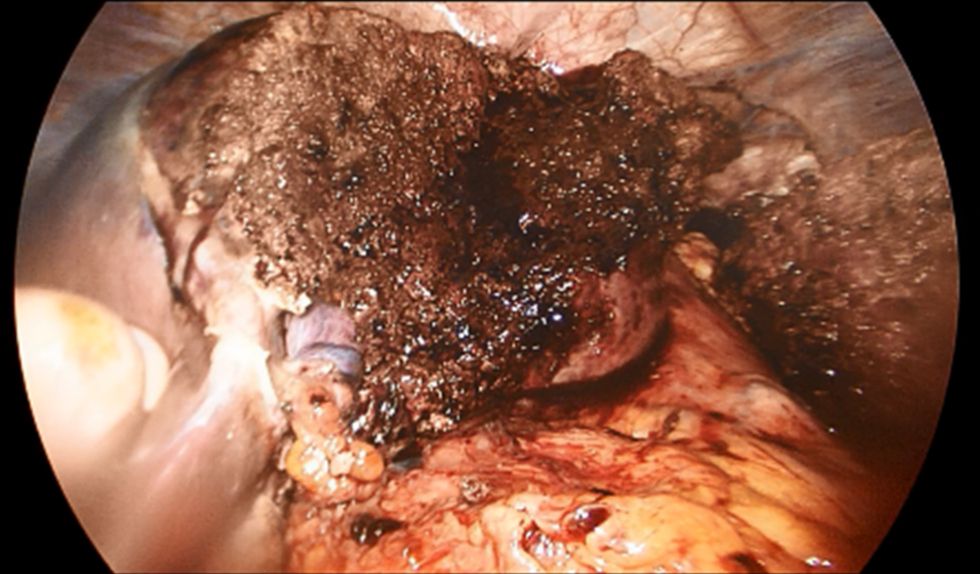
Complete left lateral sectionectomy

In total, data of 133 patients who underwent SILL were obtained. The majority of procedures were left lateral sectionectomies (65, 48.9%), followed by wedge resections (41, 30.8%) for mainly malignant lesions (73, 55.3%) The mean operative time was 133.2 min, the average estimated blood loss was 147.6 ml. The mean LOS was 4.7 days. Table 2 (Tab. 2) shows a detailed overview of all analyzed factors. Figure 2 (Fig. 2), Figure 3 (Fig. 3), Figure 4 (Fig. 4), and Figure 5 (Fig. 5) show the placement of the SILS trocar including laparoscopic instruments and various steps of the dissection of the liver parenchyma, respectively. 

### Morbidity and mortality

Overall, 9 complications were reported in 133 cases (6.8%). There were 2 cases of bile leakage, one shoulder pain, one minor allergic reaction, one transient liver failure, 2 cases of postoperative hemorrhage, one wound infection and one incisional hernia. In 10 of 15 studies, no complications appeared [19], [20], [21], [22], [24], [25], [28], [29], [30], [31]. Perioperative mortality was zero in all studies except one. One patient died of cardiac failure due to an unknown aortic valve stenosis [27].

### Conversion rate

Conversion to either multiport laparoscopic or open liver resection was necessary in three studies. Shetty and colleagues had to convert to multiport laparoscopy in 2 of 24 cases due to limitations in the length of instruments (CUSA and Harmonic ACE). Conversion to open surgery was performed in 2 cases due to major intraoperative bleeding and in two cases due to poor tumor localisation [23]. Hu and colleagues reported conversion to multiport laparoscopy in one patient due to CO2 leakage [22]. Zhao had to convert to conventional laparoscopy because of a poor visual field with a 0° laparoscopy and compromised laparoscopic manipulation, respectively [32].

### Comparison of MILL and SILL

There were two studies comparing the two minimally invasive approaches. Hu and colleagues reported good results for both techniques in terms of complications and perioperative data such as blood loss, operation time, postoperative pain and radicality [22]. 

Aldrighetti et al. found similar results for both groups as well [27]. The cost analysis showed no differences in total costs between multiport laparoscopic liver resection and SILL, whereas a significant reduction in LOS was found in the SILL group [22]. 

### Follow-up and oncological outcome

A follow-up analysis was available in 7 studies [17], [20], [22], [23], [24], [28], [29], [32]. In a 6 month follow-up period, Hu et al. found no tumor recurrence, the cosmetic outcome was favorable in all patients, there were no differences between the SILL and the multiport group with regards to quality of life [22]. The study of Chang et al. showed no tumor recurrence in 3 of 3 cases during 4–7 months [24].

Wu and colleagues found a disease-free survival in 15 of 17 cases during follow-up (6–42 months) [17]. Shetty et al. described tumor recurrence in 4 patients, all recurrences appeared within 5 months after the surgery, one patient died after 8 months due to extensive tumor progression, the exact follow-up period is not available [23]. Zhao et al. had a mean follow-up period of 5.2 months. During this time, there was no tumor recurrence as well as short- and long-term wound-site complications [32]. The examination of Weiss et al. showed a disease-free survival of 91% and 70% at 6 and 12 months, respectively. The 12-month survival was 100% [28]. 

After a median follow-up period of 12 months, there was no incisional hernia, the cosmetic result was favorable in all cases in the examination of Machado et al. [20]. Another study showed no wound pain or impairment of liver function after 2 weeks [29]. 

## Discussion

This is the first systematic review on SILL procedures including prospective trials. The present review was performed to collect the evidence on the field SILL. Altogether, 15 eligible studies with 133 patients could be identified. These studies showed good perioperative results with regards to operative time, blood loss and LOS. Free resection margins could be achieved in all but one patient. The procedure showed to be feasible in most cases, even in major resections (two hemihepatectomies).

In contrast to the few reports on SILL, there are numerous studies examining conventional MILL [33], [34], [35], [36], [37], [38], [39], [40], [41], [42], [43], [44], [45], [46], [47], [48], [49]. Among the reviewed SILL studies, intraoperative blood loss was 100 ml or less in 8 of 15 studies [18], [20], [21], [22], [28], [29], [31], [32] and LOS was shorter than 5 days in 8 studies as well [18], [19], [20], [21], [22], [24], [32], [50]. In most examinations on MILL, there are higher values for both LOS and blood loss [33], [35], [36], [38], [41], [43], [46]. On the other hand, some studies show findings that are well comparable to the results of the SILL studies [45], [47]. The overall rate of complications was low, in total 9 complications appeared in 133 patients (6.8%). In 10 studies, no complications were reported [19], [20], [21], [22], [24], [25], [28], [29], [30], [31]. In the current literature, the reports on complications after MILL procedures differ strongly. The complication rates vary between 2% and 46% [33], [34], [35], [36], [38], [41], [43], [44], [46].

However, the comparison of these results has to be done with caution since these studies are heterogenous with regards to the type of liver resection, sample size and study protocol (prospective vs. retrospective).

Conversion from SILL to either open surgery or MILL was needed in few cases, mainly due to technical problems. The conversion rate from MILL to open surgery ranges between 0 and 36% [33], [34], [36], [38], [43], [44], [46], [48], depending on the respective study. 

Most authors reported excellent cosmetic results after SILL, incisional hernia was reported in one case only. However, a follow-up of the patients was merely available in 7 of 15 studies, the follow-up period varied strongly from 2 weeks to 40 months. Due to the limited availability of data on the follow-up of these patients and a heterogenous study collective with regards to tumor entity, it is difficult to assess the oncological safety and problems with the incision site in the long-term. Similarly, follow-up data are available in some [35], [38], [41], [43], [46] but not in all studies [36], [47].

A cost analysis for SILL vs. MILL was performed in the examination by Hu et al. only [22], the total medical costs were about 2,300 USD for both procedures. Packiam et al. compared MILL to robotic liver resection, the calculated costs for MILL were much higher compared to the findings of Hu et al. (4,400 USD) [47].

Altogether, the interpretation of the results of SILL procedures remains difficult since the minority of the SILL studies had a control group with only one randomized controlled clinical trial. 

In our institution, we perform MILL for liver resections up to hemihepatectomies for both benign and malignant liver pathologies. Moreover, we have started to carry out wedge resections, segmentectomies and left lateral sectionectomies in SILL in selected cases with good preliminary results regarding feasibility, clinical outcome and cosmetic satisfaction. 

## Conclusion

In summary, there are some potential benefits of the SILL procedure compared to multiport laparoscopy such as reduced LOS or cosmetic result. However, the review of the current literature reveals, that a broad evidence on this topic is missing and that further prospective, randomized controlled trials are urgently needed.

## Notes

### Competing interests

The authors declare that they have no competing interests.

## Figures and Tables

**Table 1:**
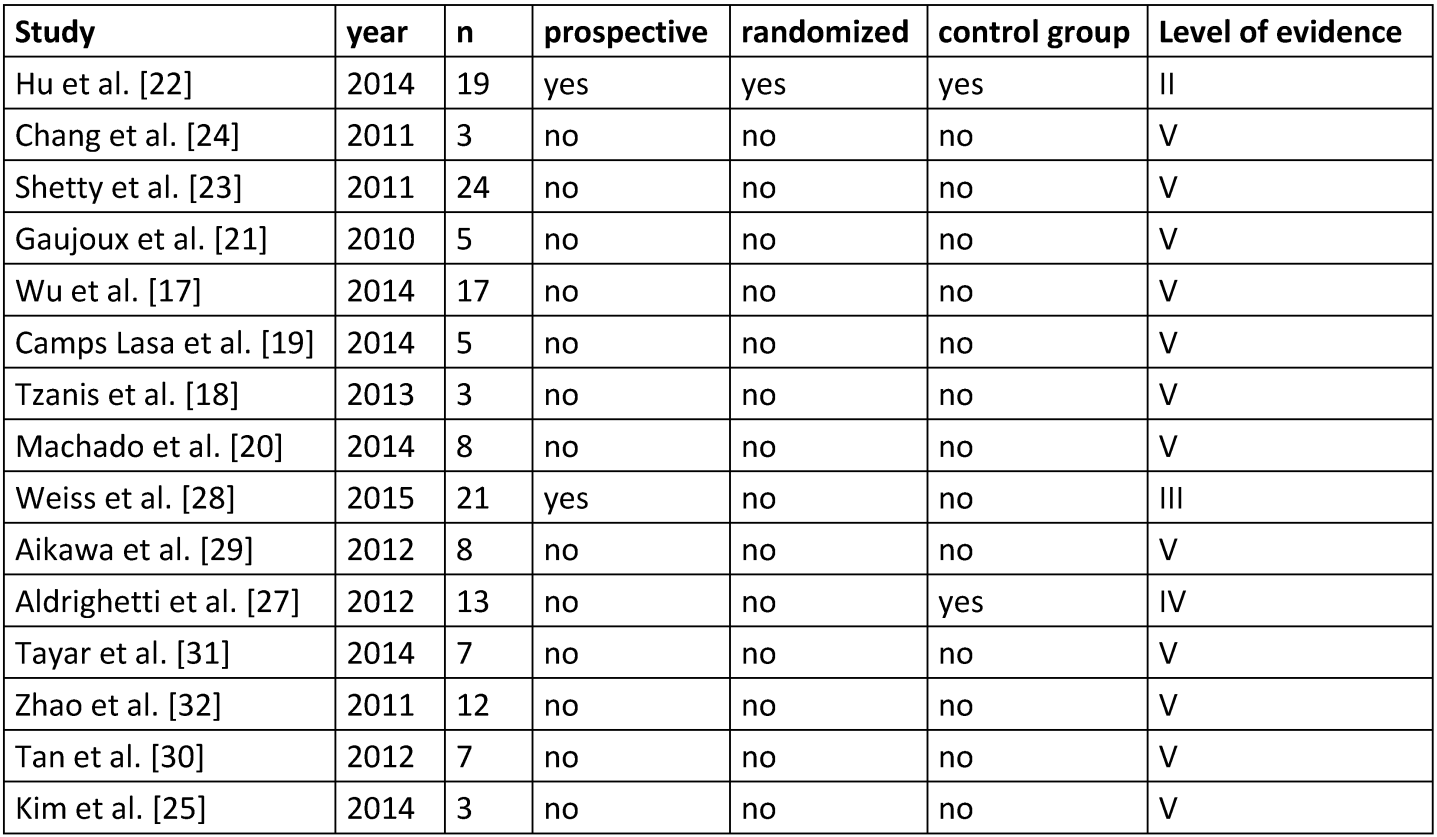
Quality criteria of the included studies

**Table 2:**
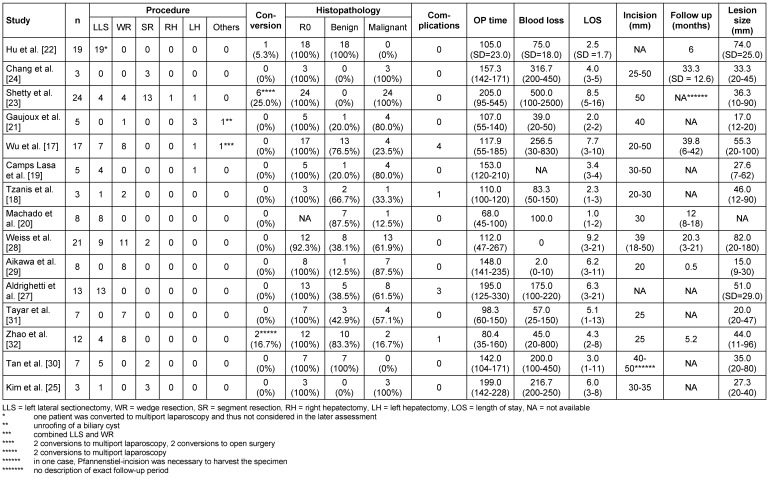
Characteristics of the reviewed studies
